# Supplementary material for: A cross-sectional study evaluating insulin injection techniques and the impact of instructions from various healthcare professionals on insulin users in the southern region of Saudi Arabia
Source: PeerJ. 2025 May 12;13:e19394. doi: 10.7717/peerj.19394 (PMC12080473; doi:10.7717/peerj.19394)
Supplement: Supplemental Information 2 [file peerj-13-19394-s002.pdf]

STROBE Statement—Checklist of items that should be included in reports of ***cross-sectional studies***

	Item No	Recommendation	Remark
**Title and abstract**	1	(*a*) Indicate the study's design with a commonly used term in the title or the abstract	The study design is indicated in the lines 1 and 37
		(*b*) Provide in the abstract an informative and balanced summary of what was done and what was found	The balanced summary of "what was done and what was found' is mentioned in the lines 37-39 and 44-49
**Introduction**			
Background/rationale	2	Explain the scientific background and rationale for the investigation being reported	The scientific background is written in the lines 71-82; the rationale for the current study is in 83-89.
Objectives	3	State specific objectives, including any prespecified hypotheses	This point is indicated in the lines 89-92.
**Methods**			
Study design	4	Present key elements of study design early in the paper	The key elements of the study design have been mentioned in the lines95-96, 102, 104-105, and 119-122.
Setting	5	Describe the setting, locations, and relevant dates, including periods of recruitment, exposure, follow-up, and data collection	The period of the study is indicated in lines96-97; location where the study was conducted is mentioned in 119-120. The administration of the questionnaire, follow-up and the data collection are mentioned in lines 124-126.
Participants	6	(*a*) Give the eligibility criteria, and the sources and methods of selection of participants	The eligibility criteria to choose the participants are described in the lines 120-122, and the method of selection of the participants is described in line # 124.
Variables	7	Clearly define all outcomes, exposures, predictors, potential confounders, and effect modifiers. Give diagnostic criteria, if applicable	The study outcomes that are to be measured are given in the lines 108-117.
Data sources/ measurement	8*	For each variable of interest, give sources of data and details of methods of assessment (measurement). Describe comparability of assessment methods if there is more than one group	The source of data is the participants' responses collected using the questionnaire (line# 124-126), and the method of assessment and the comparability of the groups

			are mentioned in lines 126-132.
Bias	9	Describe any efforts to address potential sources of bias	Not applicable
Study size	10	Explain how the study size was arrived at	The sample size calculation is described in the lines 97-100.
Quantitative variables	11	Explain how quantitative variables were handled in the analyses. If applicable, describe which groupings were chosen and why	The method for handling the quantitative variables is described in the lines 128-131.
Statistical methods	12	(*a*) Describe all statistical methods, including those used to control for confounding	There are no confounding factors in this study and the statistical method used is described in the lines 128-132.
		(*b*) Describe any methods used to examine subgroups and interactions	Not applicable.
		(*c*) Explain how missing data were addressed	The responses with missing data were eliminated from the data analysis.
		(*d*) If applicable, describe analytical methods taking account of sampling strategy	Not applicable.
		(*e*) Describe any sensitivity analyses	Not applicable.
**Results**			
Participants	13*	(a) Report numbers of individuals at each stage of study—eg numbers potentially eligible, examined for eligibility, confirmed eligible, included in the study, completing follow-up, and analysed	The number of participants at each stage is described in the lines 137-138.
		(b) Give reasons for non-participation at each stage	Incomplete response (line 138)
		(c) Consider use of a flow diagram	Since there are no complex methods of recruitment, flow diagram may not be necessary.
Descriptive data	14*	(a) Give characteristics of study participants (eg demographic, clinical, social) and information on exposures and potential confounders	The charactiestics of the study participants are given in the lines 139-147.
		(b) Indicate number of participants with missing data for each variable of interest	The responses with missing data were eliminated from the data analysis (line# 137-138)
Outcome data	15*	Report numbers of outcome events or summary measures	All the outcome events and summary measures are explained in the lines 148-203

Main results	16	(*a*) Give unadjusted estimates and, if applicable, confounder-adjusted estimates and their precision (eg, 95% confidence interval). Make clear which confounders were adjusted for and why they were included	Not applicable
		(*b*) Report category boundaries when continuous variables were categorized	Not applicable
		(*c*) If relevant, consider translating estimates of relative risk into absolute risk for a meaningful time period	Not applicable
Other analyses	17	Report other analyses done—eg analyses of subgroups and interactions, and sensitivity analyses	Not applicable
**Discussion**			
Key results	18	Summarise key results with reference to study objectives	The key results are compared with the existing relevant studies in the lines 211-300
Limitations	19	Discuss limitations of the study, taking into account sources of potential bias or imprecision. Discuss both direction and magnitude of any potential bias	The limitations of the study is discussed in the lines 302-304
Interpretation	20	Give a cautious overall interpretation of results considering objectives, limitations, multiplicity of analyses, results from similar studies, and other relevant evidence	The interpretations of the key results are explained in the lines 211-300
Generalisability	21	Discuss the generalisability (external validity) of the study results	Discussed in the lines 302-304.
**Other information**			
Funding	22	Give the source of funding and the role of the funders for the present study and, if applicable, for the original study on which the present article is based	The funding information is mentioned in the lines 315-317.

*Give information separately for exposed and unexposed groups.

Note: An Explanation and Elaboration article discusses each checklist item and gives methodological background and published examples of transparent reporting. The STROBE checklist is best used in conjunction with this article (freely available on the Web sites of PLoS Medicine at http://www.plosmedicine.org/, Annals of Internal Medicine at http://www.annals.org/, and Epidemiology at http://www.epidem.com/). Information on the STROBE Initiative is available at www.strobe-statement.org.
