# Supplementary material for: A cross-sectional study evaluating insulin injection techniques and the impact of instructions from various healthcare professionals on insulin users in the southern region of Saudi Arabia
Source: PeerJ. 2025 May 12;13:e19394. doi: 10.7717/peerj.19394 (PMC12080473; doi:10.7717/peerj.19394)
Supplement: Supplemental Information 3 [file peerj-13-19394-s003.pdf]

تقييم تقنيات حقن الأنسولين (IIT) بين مرضى السكري في مركز الرعاية الثالثية ، منطقة عسير ، المملكة العربية السعودية.

أعزائي المجيبين المحتملين،نجري بحثًاحول تقييم تقنيات حقن الأنسولين بين مرضى السكري في منطقة عسير،المملكة العربية السعودية . لذلك،أطلب دعمكم في هذا المشروع وأطلب منك أن تتفضل بالمشاركة في هذا الاستبيان .
علاوة على ذلك،يجب الحفاظ على سرية جميع المعلومات الشخصية الواردة في هذا الاستبيان.
لذلك، ستظل إجاباتك آمنة. لن يتمكن أحد من التعرف عليك أو على إجاباتك، ولن يعرف أحد ما إذا كنت قد شاركت في الدراسة

- ð
  يوافق
- ð
  لا اوافق

* Indicates required question

1. العمرAge (سنه) *

   ____

2. الجنسSex *

   Mark only only one oval.

   ◯ Maleذكر
   ◯ Femaleأنثى

3. Educational statusالمستوى التعليمي *

   Mark only one oval.

   ◯ Illiterate/Uneducated(امي) غير متعلم
   ◯ Student طالب
   ◯ Bachelor degree درجه البكالوريس
   ◯ Post graduate and above الدرسات العليا وما فوق

4. Area of residence مكان الاقامه *

Mark only one oval.

- Urban مدينه
- Rural قرية

5. Occupation المهنة *

Mark only one oval.

- Employed موظف
- Unemployedغير موظف
- College Student طالب جامعي
- School student طالب مدرسه
- Other: _______________

6. Insulin administrationطريقه الحقن *

Mark only one oval.

- Self بنفسك
- By others بمساعدة الاخرين

7. Type of diabetes نوع السكري *

Mark only one oval.

- Type 1 diabetesالسكري النوع الأول
- Type 2 diabetesالسكري النوع الثاني
- Gestational diabetes سكر الحمل

8. Duration of diabetes (سنه) مدة المرض بالسنوات *

____

9. Do you think your insulin injection technique is correct? هل تعتقد أن طريقة حقن الأنسولين لديك صحيحة؟ *

Mark only one oval.

- Yes نعم
- Not sure لست متأكدا

10. Who instructed you about insulin injection technique?من الذي دربك على حقن الأنسولين *

Mark only one oval.

- Clinician / Physician الطبيب
- Diabetes educator طبيب السكري
- Pharmacist صيدلي
- Nurse ممرض
- Not received any training لم اتلقَ أي تدريب
- Other: ____________________

11. Where do you store your insulin pen after you begin to use the pen? أين تخزن قلم الأنسولين بعد البدء في استخدام القلم؟ *

Mark only one oval.

- Refrigerator ثلاجة
- Room temperature درجة حرارة الغرفة

12. Before the injection do you clean the skin with disinfectant (e.g. an alcohol swab)? قبل الحقن،هل تقوم بتنظيف الجلد "مكان الحقن" بمطهر (على سبيل المثال باستخدام مسحة طبية)؟ *

Mark only one oval.

- Yes نعم
- No لا

13. Before attaching the needle into the pen, do you clean the stopper with disinfectant (e.g. an alcohol swab)? قبل توصيل الإبرة في القلم ،هل تقوم بتنظيف السدادة بمطهر (على سبيل المثال باستخدام مسحة طبية)? *

Mark only one oval.

- Yes نعم
- No لا

14. Do you check the expiry date of your pen? هل تحقق من تاريخ انتهاء قلمك؟ *

Mark only one oval.

- Yes نعم
- No لا

15. Do you bring insulin to room temperature before injecting it?هل تجلب الإبرة إلى درجة حرارة الغرفة قبل حقنها ؟ *

Mark only one oval.

- Yes نعم
- No لا

16. If you use cloudy insulin, do you re-mix your insulin before use? *
إذا كنت تستخدم الأنسولين الغائم، هل تعيد خلط/تحريك الإنسولين قبل الاستخدام؟

Mark only one oval.

- Yes نعم
- No لا

17. Do you use new needle for every injection? هل تستخدم إبرة جديدة لكل حقنه؟ *

Mark only one oval.

- Yes نعم
- No لا

18. Do you prime the device by observing drop of insulin at needle tip?هل أنت تراقب القلم من خلال مراقبة قطرات الأنسولين في طرف الإبرة؟ *

Mark only one oval.

- Yes نعم
- No لا
- I have no idea on priming ليس لدي أي فكرة

19. What is the gap between injection and meal?كم الوقت الذي تجعله بين الحقن والوجبة؟ *

Mark only one oval.

- 0-15 15-0 دقيقة minutes
- 16-30 30-16 دقيقة minutes
- More than 30 minutes أكثر من 30 دقيقه

20. What injection sites do you use (tick all relevant answers)? مامواقع الحقن التي تستخدمها(ضع علامة على جميع الاجابات ذات الصلة)؟ *

Check all that apply.

- [ ] Abdomen البطن
- [ ] Thigh الفخذ
- [ ] Buttocks الارداف
- [ ] Arm الذراع

21. What site do you use the most? ما هو الجزء اللي تحقن فيه أكثر؟ *

Mark only one oval.

- Abdomen البطن
- Thigh الفخذ
- Buttocks الارداف
- Arm الذراع

22. Do you rotate injection sites? هل تغير مكان الحقن *

Mark only one oval.

- Yes نعم
- No لا

23. What is the angle of insulin pen while penetrating into the skin? *
ما هي زاوية القلم أثناء الحقن ؟

Mark only one oval.

- 60 60درجهdegree
- 90 90درجهdegree
- 45 45درجهdegree
- Don't know لا أعرف

24. Do you make a skin fold? هل تطوي الجلد عند الحقن؟ *

Mark only one oval.

- Yes نعم
- No لا

25. Are your injection sites inspected in each visit?هل يتم فحص مواقع الحقن الخاصة بك في كل زيارة للمستشفى؟ *

Mark only one oval.

- Yes نعم
- No لا

26. Do you have any swelling or lumps under the skin at your usual injection sites that have been there for some time? هل لديك أي تورم أو كتل تحت الجلد في مواقع الحقن المعتادة لديك؟ *

Mark only one oval.

- Yes نعم
- No لا

27. How long do you leave the needle under the skin after you have pushed the plunger in? كم من الوقت تترك الإبرة تحت الجلد بعد الحقن؟ *

Mark only one oval.

- less than 5 sec اقل من 5 ثواني
- 5 – 10 sec من 5 الى 10 ثواني
- 10 10 ثواني seconds
- I remove the needle immediately ازيل الإبرة على الفور

28. Does insulin ever leak out of your injection site on the skin? هل يتسرب الأنسولين من موقع الحقن على الجلد؟ *

Mark only one oval.

- Yes نعم
- No لا

29. Do you message the site of injection after injection? هل تدلك موقع الحقن بعد الحقن؟ *

Mark only one oval.

- YES نعم
- NO لا

30. Do you ever inject through your clothing? هل تحقن الانسولين من خلال ملابسك؟ *

Mark only one oval.

- Yes نعم
- No لا

31. How long do you use an insulin pen after first use? كم مدة إتستخدام قلم الأنسولين بعد أول استخدام؟ *

Mark only one oval.

- One month شهر واحد
- More than 1 month أكثر من شهر واحد

32. In the last six months have you experienced hypoglycemia (low blood sugar)? *
في الأشهر الستة الماضية ، هل عانيت من انخفاض في سكر الدم؟

Mark only one oval.

- Yes نعم
- No لا

33. If yes, how many times in the last six months have you had hypoglycemia so severe you needed assistance from another person? إذا كانت الإجابة بنعم ، *
كم مرة خلال الأشهر الستة الماضية كنت تعاني من نقص السكر في الدم لدرجة أنك كنت بحاجة إلى مساعدة من شخص آخر؟

Mark only one oval.

- None لايوجد
- 1 -times 2 مره الى مرتين
- 3- times 5 ثلاث مرات الى خمس مرات
- More than 5 times أكثر من خمس مرات

34. Do you have your own glucometer? هل تملك جهاز قياس السكر خاص بك؟ *

Mark only one oval.

- Yes نعم
- No لا

35. How often do you do finger-pricks to check your blood glucose? *
كم عدد المرات التي تقيس فيها السكر ؟

Mark only one oval.

- More than 4 times a day اكثر من 4 مرات في اليوم
- 3 4-3 الى 4 مرات في اليوم times
- 1 2-1 الى 2 مرتين في اليوم day a times
- Several times a week عدت مرات في الاسبوع
- I rarely or never check blood glucoseنادرا ما اقيس او لا أقيس ابدا

36. Do you ever miss or skip an injection? هل تخطيت جرعه من قبل؟ *

Mark only one oval.

- ( ) Always دائما
- ( ) Sometimes أحيانا
- ( ) Rarely نادرا
- ( ) Never ابدا

37. Reason for missing a dose?(more than one answer are allowed) *
ما الأسباب التي جعلتك تتخطى الجرعه (يسمح بأكثر من إجابه)؟

Check all that apply.

- [ ] Too busy مشغول جدا
- [ ] Forgetfulness نسيت
- [ ] Skipped meal تخطي وجبة
- [ ] Traveling or changing normal routine السفر أو تغييرالروتين العادي
- [ ] Embarrassing to inject in publicمحرجة للحقن في الأماكن العامة
- [ ] Other: ____________________

38. Do you have any challenges / difficulties in using insulin?(more than one answer are allowed) *
هل لديك أي تحديات / صعوبات في استخدام الأنسولين؟ (يُسمح بأكثر من إجابة)

Check all that apply.

- [ ] Preparing insulin pen and injection site تحضير قلم الأنسولين وموقع الحقن
- [ ] Taking injection on time أخذ الحقن في الوقت المحدد
- [ ] Taking injection during busy hours أخذ الحقن خلال ساعات العمل
- [ ] Pain associated with injection الألم المرتبط بالحقن
- [ ] Taking injection away from home, during vacation, or during business trip. أخذ الحقن بعيدًا عن المنزل أوأثناء الإجازة أو أثناء رحلة العمل.
- [ ] Number of daily injections عدد الحقن اليومية
- [ ] Regimen is too complicated نظام معقد للغاية
- [ ] Adjusting insulin doses ضبط جرعات الأنسولين

This content is neither created nor endorsed by Google.

Google Forms
