# Supplementary material for: A cross-sectional study evaluating insulin injection techniques and the impact of instructions from various healthcare professionals on insulin users in the southern region of Saudi Arabia
Source: PeerJ. 2025 May 12;13:e19394. doi: 10.7717/peerj.19394 (PMC12080473; doi:10.7717/peerj.19394)
Supplement: Supplemental Information 4 [file peerj-13-19394-s004.pdf]

(سنه) Age العمر

Sex الجنس Educational status ى التعليمي Area of residence كان الاقامه Occupation المهنة

Insulin administrationه الحقن	Type of diabetes نوع السكري	Duration of diabetes (سنه)	Do you think your insulin ir	Who instructed you about
	1- Type 1 DM	1- 1 to 10 years	1- Yes	1- Clinician / Physician
	2- Type 2 DM	2- 11 to 20 years	2- Not sure	2- Diabetes educator
	3- Gestational DM	3- 21 to 30 years		3- Pharmacist
		4- 31 to 50 years		4- Nurse
				5- Not received any trainin

Where do you store your i	Before the injection do you	Before attaching the needl	Do you check the expiry da	Do you bring insulin to roo
1- Refregirator	1- Yes	1- Yes	1- Yes	1- Yes
2- Room temperature	0- No	0- No	0- No	0- No

g

If you use cloudy insulin, d	Do you use new needle fo	Do you prime the device b	What is the gap between i	What site do you use the r
1- Yes	1- Yes	0- No	1- 0 to 15 minutes	1- Abdomen
0- No	0- No	1- Yes	2- 16 to 30 minutes	2- Thigh
		2- Ihave no idea on priming	3- more than 30 minutes	3- Buttocks
				4- Arm

Do you rotate injection site	What is the angle of insulin	Do you make a skin fold?	Are your injection sites ins	Do you have any swelling
1- Yes	1- 45 degree	1- Yes	1- Yes	1- Yes
0- No	2- 60 degree	0- No	0- No	0- No
	3- 90 degree			
	Don’t know			

How long do you leave the	Does insulin ever leak out	Do you message the site c	Do you ever inject through	How long do you use an in
1- < 5sec	1- No	1- No	1- No	1- One month
2- 5 to 10 sec	0- Yes	0- Yes	0- Yes	2- Morethan one month
3- > 10 sec				
4- I remove the needle immediately				

In the last six months have	If yes, how many times in t	Do you have your own glu	How often do you do finge	Do you ever miss or skip a
1- No	1- 1 to 2 times	1- Yes	1- 1 to 2 times a day	1- Never
0- Yes	2- 2-5 times	0- No	2- 3 to 4 times a day	2- Rarely
	3- Morethan 5 times		3- Morethan 4 times a day	3- Sometimes
			4- several times a week	4- Always
			5- I rarely or never check	

Reason for missing a dose	Do you have any challenges / difficulties in using insulin?(more than one answer are allowed) سمح بأكثر من إجابة)
1- Too busy	1- Preparing insulin pen and injection site
2- Forgetfullness	2- Taking injection on time
3- Skipped meal	3- Taking injection during busy hours
4- Embarrased to inject in	4- Pain associated with injection
5- Traveling or changing n	5- Taking injection away from home, during vacation, or during business trip
6- Others (Sugar level is m	6- Number of daily injections
	7- Regimen is too complicated
	8- Adjusting insulin dose
	9- Measuring blood glucose routinely
	10- Fear of weight gain
	11- Fear of low sugar

هل لديك أي تحديات / صعوبات في استخدام الأنسولين؟ (يُ
